# Clinical course and risk factors for recurrence of positive SARS-CoV-2 RNA: a retrospective cohort study from Wuhan, China

**DOI:** 10.18632/aging.103795

**Published:** 2020-09-10

**Authors:** Jie Chen, Xiaoping Xu, Jing Hu, Qiangda Chen, Fengfeng Xu, Hui Liang, Nanmei Liu, Hengmei Zhu, Jinlong Lan, Lan Zhou, Jiajun Xing, Ning Pu, Zhigang Cai

**Affiliations:** 1 Department of Cardiothoracic Surgery, Naval Medical Center of PLA, Shanghai 200052, People’s Republic of China; 2 Department of Infectious Disease, Guanggu Branch of Hubei Province Maternity and Childcare Hospital, Hubei 430073, People’s Republic of China; 3 Department of General Surgery, Zhongshan Hospital, Fudan University, Shanghai 200032, People’s Republic of China; 4 Department of Special Treatment, Eastern Hepatobiliary Surgery Hospital, Naval Military Medical University, Shanghai 200438, People’s Republic of China

**Keywords:** coronavirus disease 2019, COVID-19, SARS-CoV-2, recurrence, clinical course, risk factor

## Abstract

The coronavirus disease 2019 (COVID-19) pandemic is caused by the severe acute respiratory syndrome coronavirus 2 (SARS-CoV-2). The objective of this study was to determine the clinical course and risk factors for patients showing recurrent SARS-CoV-2 RNA positivity. A total of 1087 COVID-19 patients confirmed by RT-PCR from February 24, 2020 to March 31, 2020 were retrospectively enrolled. Advanced age was significantly associated with mortality. In addition, 81 (7.6%) of the discharged patients tested positive for SARS-CoV-2 RNA during the isolation period. For patients with recurrent RT-PCR positivity, the median duration from illness onset to recurrence was 50 days. Multivariate regression analysis identified elevated serum IL-6, increased lymphocyte counts and CT imaging features of lung consolidation during hospitalization as the independent risk factors of recurrence. We hypothesized that the balance between immune response and virus toxicity may be the underlying mechanism of this phenomenon. For patients with a high risk of recurrence, a prolonged observation and additional preventative measures should be implemented for at least 50 days after illness onset to prevent future outbreaks.

## INTRODUCTION

The coronavirus disease 2019 (COVID-19) pandemic is caused by the severe acute respiratory syndrome coronavirus 2 (SARS-CoV-2) [1, 2]. As of April 18, 2020, 2,121,675 confirmed cases of COVID-19 and 142,299 related deaths have been reported from 213 countries according to the World Health Organization (WHO) [3]. Although several studies have summarized the epidemiological and clinical features of SARS-CoV-2 infection [4–6], and research is going on viral pathogenicity and mechanism. However, the exact origin of SARS-CoV-2 is controversial and a potential threat to a new outbreak [7, 8]. Furthermore, little is known regarding the immune response against SARS-CoV-2 infection, which in turn makes it difficult to assess complete recovery with no further risk of infection. The latter is a crucial factor in “flattening the curve” of COVID-19 and preventing additional outbreaks.

In the early stages of the COVID-19 outbreak that was located to Wuhan, China, the severe shortage and limitations in the detection and accuracy of the RT-PCR test restricted identification of infected patients. The diagnostic techniques have improved substantially since [9], and two or more multipoint throat-swabs are taken over 24 hours apart prior to discharge in order to minimize the false negative rate of RT-PCR tests [10]. Lan L et al. [11] reported that four medical professionals with COVID-19 who met the criteria for hospital discharge (including two consecutive negative RT-PCR results) reverted to SARS-CoV-2 positivity, indicating a potential asymptomatic carrier state. It remains to be determined whether patients with recurrent SARS-CoV-2 RNA positivity remain infectious after discharge. Furthermore, the clinical and radiological characteristics of the COVID-19 patients with recurrence is largely unknown.

Herein, we retrospectively analyzed 1087 patients with confirmed COVID-19 and explored the clinical course and risk factors of SARS-CoV-2 RNA recurrence by RT-PCR during post-discharge isolation.

## RESULTS

### Clinico-demographic characteristics of patients

A total of 1087 consecutive COVID-19 pneumonia patients positive for SARS-CoV-2 RNA were enrolled in this study. The median age of the cohort was 60 years (9 to 100 years; IQR - 49-69 years) and 635 (58.4%) of the patients were women. The majority (83.1%) of the cases were mild, whereas the proportion of severe and critical cases were 13.2% and 3.7% respectively. Most patients (874, 80.4%) had bilateral pulmonary infiltration on the chest CT, while 730 (67.2%) and 525 patients (48.3%) respectively showed ground-glass appearance and consolidation. In addition, 887 out of 1007 (88.1%) patients were positive for serum IgG, while 797 out of 1057 (75.4%) patients were positive for serum IgM against COVID 19.

The median length of hospitalization was 12 days (1-38 days; IQR, 8-17 days), and 20 patients died during hospitalization whereas 1067 were discharged. The total mortality rate was 1.8% and the discharge rate was 98.2%. Among the fatalities, 5 patients were graded as severe with mortality rate of 3.5%, and 15 were critical cases with a high mortality rate of 37.5%. The total mortality rate of the severe and critical cases was 10.6%. The median age of the deceased patients was 83 years (65 to 92 years; IQR, 79.3-87.8 years), which was significantly higher than that of the discharged patients (P<0.001). The main causes of deaths were multiple organ failure (MOSF), most commonly affecting the lungs, heart, liver and kidneys. Other clinical features, laboratory examinations and imaging findings are summarized in Supplementary Table 1.

### Characteristics of patients with SARS-CoV-2 RNA recurrence

Eighty-one (7.6%) of the discharged patients reverted to SARS-CoV-2 RNA positive after two negative RT-PCR tests during the post-discharge isolation period. The median age of the recurring cases was 62 years (range 16-90 years; IQR, 50.5-68 years), and 51 (63.0%) were female. Twenty (24.7%) patients had accompanying hypertension and 9 (11.1%) had diabetes. Furthermore, 84.0% (68), 14.8% (12) and 1.2% (1) of the cases were mild, severe and critical respectively. Most of these patients had the initial symptoms of COVID-19 infection prior to positive SARS-CoV-2 RNA diagnosis, and only 15 (18.5%) were asymptomatic when first diagnosed. Before hospitalization, pulmonary infection was confirmed in 70 patients via CT scan, and 65 (65.3%) received anti-viral agents.

Laboratory and CT imaging results from the inpatient hospital-stay are summarized in Table 1. Seven (8.6%) patients had lymphocytopenia and only 4 (4.9%) patients had neutrophilia. High-sensitivity CRP was elevated in 8 (9.9%) patients, and the ESR, procalcitonin and IL-6 levels were increased in 27 (33.3%), 14 (17.3%) and 11 (13.6%) patients. Furthermore, 10 (12.3%) patients developed liver injury with elevated ALT, 4 (4.9%) demonstrated myocardial damage with elevated Accu-Tell troponin, and 11 (13.6%) patients had kidney injury with elevated serum BUN and creatinine levels. CT images revealed consolidation, ground-glass opacity and bilateral pulmonary infiltration in 49 (60.5%), 56 (69.1%) and 70 (86.4%) patients, respectively. Finally, 72 of 77 (93.5%) patients were positive for serum IgG, whereas 68 of 79 (86.1%) were positive for serum IgM against COVID-19.

**Table 1:** Clinico-demographic characteristics of patients with recurrence of SARS-CoV-2 RNA positivity.

**Variables**	**No. (n=81)**	**Percentage (%)**
General features		
*Clinical severity of disease*		
Mild	68	84.0%
Severe	12	14.8%
Critical	1	1.2%
*Age*		
Median (IQR)	62.0 (50.5-68.0)	
*Gender*		
Male	30	37.0%
Female	51	63.0%
*Hypertension*		
Yes	20	24.7%
No	61	75.3%
*Diabetes*		
Yes	9	11.1%
No	72	88.8%
Illness onset
*Fever*		
Yes	41	50.6%
No	40	49.4%
*Cough*		
Yes	44	54.3%
No	37	45.7%
*Chest congestion*		
Yes	18	22.2%
No	63	77.8%
*Weak*		
Yes	34	42.5%
No	46	57.5%
*Muscular soreness*		
Yes	15	18.5%
No	66	81.5%
*Pulmonary infection (CT)*		
Yes	70	86.4%
No	5	6.2%
Unknown	6	7.4%
*Anti-virus therapy*		
Yes	53	65.4%
No	15	18.5%
Unknown	13	16.0%
In hospital		
*Fever*		
Yes(>=37.3 °C once or more)	18	22.2%
No	63	77.8%
*Internal visceral dysfunctions*		
Yes	30	37.0%
No	51	63.0%
*Comorbid diseases*		
Yes	46	56.8%
No	35	43.2%
*White blood cell count, ×10^9^ per L*		
<4	6	7.4%
4-10	71	87.7%
>10	3	3.7%
Unknown	1	1.2%
*Neutrophil count, ×10^9^ per L*		
<1.8	3	3.7%
1.8-6.3	73	90.1%
>6.3	4	4.9%
Unknown	1	1.2%
*Lymphocyte count, ×10^9^* *per L*		
<1.1	7	8.6%
1.1-3.2	72	88.9%
>1.1	1	1.2%
Unknown	1	1.2%
*Platelet count, ×10^9^* *per L*		
<125	4	4.9%
125-350	69	85.2%
>350	7	8.6%
Unknown	1	1.2%
*ALT*		
<40	71	87.7%
>=40	10	12.3%
*Albumin*		
<35	10	12.3%
>=35	71	87.7%
*C-reactive protein*		
<10	71	87.7%
>=10	8	9.9%
Unknown	2	2.5%
*ESR 30min*		
<20	13	16.0%
>=20	27	33.3%
Unknown	41	50.6%
*Procalcitonin*		
<=0.05	47	58.0%
>0.05	14	17.3%
Unknown	20	24.7%
*D-dimer*		
<0.5	47	58.0%
>=0.5	18	22.2%
Unknown	16	19.8%
*BUN*		
<=6.5	68	84.0%
>6.5	11	13.6%
Unknown	2	2.5%
*Creatinine*		
<90	68	84.0%
>=90	11	13.6%
Unknown	2	2.5%
*Accu-Tell Troponin*		
<15.6	47	58.0%
>=15.6	4	4.9%
Unknown	30	37.0%
*IL-6*		
<10	43	53.1%
>=10	11	13.6%
Unknown	27	33.3%
*IgG*		
Positive	72	88.9%
Negative	5	6.2%
Unknown	4	4.9%
*IgM*		
Positive	68	84.0%
Negative	11	13.6%
Unknown	2	2.5%
Imaging features		
*Consolidation*		
Yes	49	60.5%
No	32	39.5%
*Ground-glass opacity*		
Yes	56	69.1%
No	25	30.9%
*Bilateral pulmonary infiltration*		
Yes	70	86.4%
No	11	13.6%

### Clinical course of patients with SARS-CoV-2 recurrence

**Figure 1:**
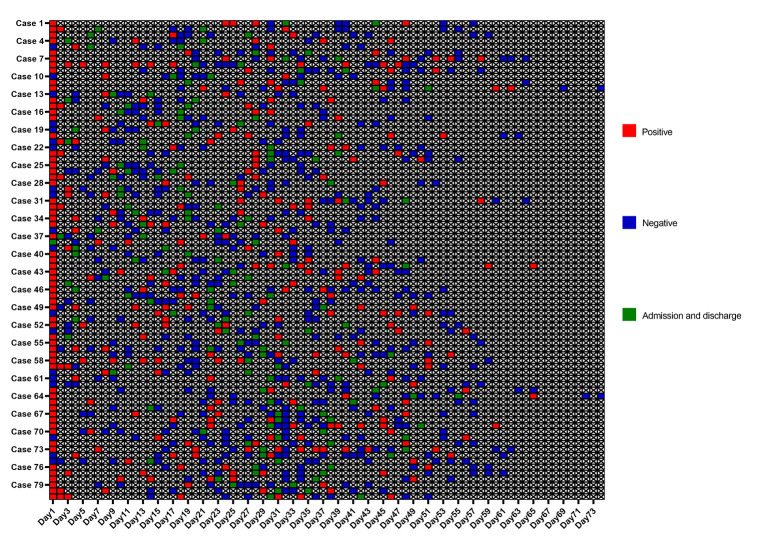
**Individual duration of viral shedding and positive SARS-CoV-2 RNA recurrence from illness onset after discharge.** The timing and results of RT-PCR examinations for SARS-CoV-2 RNA in details. SARS-CoV-2=severe acute respiratory syndrome coronavirus 2. RT-PCR=reverse transcription-polymerase chain reaction.

**Figure 2:**
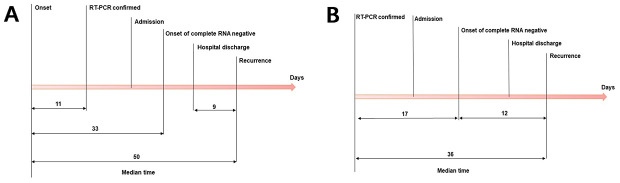
**The median duration of different stages in patients with recurrence of positive SARS-CoV-2 RNA after discharge.** (**A**) The median duration from illness onset to initial RT-PCR confirmation, onset of complete RNA negative status and recurrent RT-PCR positivity after discharge, and from discharge to recurrence. (**B**) The median duration from initial RT-PCR confirmation to onset of complete RNA negative status and recurrent RT-PCR positivity after discharge, and from onset of complete RNA negative status to recurrence. SARS-CoV-2=severe acute respiratory syndrome coronavirus 2. RT-PCR=reverse transcription-polymerase chain reaction.

As shown in Figure 1, the median length of hospitalization for patients that reverted to SARS-CoV-2 RNA positive state was 12 days (range, 4-27 days; IQR, 7-17 days). The median duration from discharge to recurrence was 9 days (range, 3-18 days; IQR, 7-10 days), and that from the onset of illness to RT-PCR confirmation was 11 days (range, 0-57 days; IQR, 1.5-21 days) (Figure 2A). In addition, the time from illness onset to complete RNA negative status was 33 days (range, 6-82 days; IQR, 20-41 days), and from illness onset to recurrence was 50 days (range, 21-95 days; IQR, 36.5-59.5 days). As shown in Figure 2B, the median duration from initial RT-PCR diagnosis to recurrence was 36 days (range, 16-64 days; IQR, 26.5-45 days). In addition, the median duration between the initial diagnostic RT-PCR and complete RNA negative status was 17 days (range, 1-45 days; IQR, 8-29 days), while that between complete RNA negative status and recurrence was 12 days (range, 4-27 days; IQR, 7-17 days).

Amongst these 81 patients, 37 (45.7%) received oxygen support. However, no invasive mechanical ventilation (IMV) or IMV with extracorporeal membrane oxygenation (ECMO) was used. The optimal antiviral therapy was administered in 69 (85.2%) patients, including arbidol hydrochloride (40 patients, 49.4%), interferon alfa (17 patients, 21.0%), entecavir/tenofovir (7 patients, 8.6%) and oseltamivir (5 patients, 6.2%). Fifty-one patients (63%) were treated with Chinese patented drugs, such as Lianhuaqingwen capsule. Vitamin C was given to 41 (50.6%) patients, and immunomodulators like thymopentin and immunoglobulin were administrated to 8 (9.9%) patients.

### Associated risk factors with recurrence of positive SARS-CoV-2 RNA

As shown in Table 2, positive SARS-CoV-2 RNA recurrence correlated positively with serum IL-6 level (P=0.010) and CT imaging depicting consolidation (P=0.031). In the univariate analysis, elevated lymphocyte count (P=0.194, OR=1.644; 95% CI, 0.776-3.484), elevated serum IL-6 (P=0.013, OR=2.504; 95% CI, 1.218-5.150), consolidation on CT imaging (P=0.033, OR=1.655; 95% CI, 1.042-2.629) and bilateral pulmonary infiltration (P=0.196, OR=1.540; 95% CI, 0.800-2.966) were identified as potential risk factors for recurrence of SARS-CoV-2 RNA positivity (Table 3). Multivariate analysis concluded that elevated lymphocyte count (P=0.038, OR=2.321; 95% CI, 1.048-5.138), serum IL-6 level (P=0.004, OR=3.050; 95% CI, 1.432-6.499) and consolidation features on CT imaging (P=0.038, OR=1.641; 95% CI, 1.028-2.620) were the independent risk factors of recurrence (Table 4).

**Table 2:** Correlations between clinical characteristics and recurrence of SARS-CoV-2 RNA positivity in discharged patients.

**Variables**	**No. (n=1067)**	**No recurrence n=986**	**Recurrence n=81**	**P value**
General features				
*Clinical severity of disease*				
Mild	903	835	68	0.684
Severe	139	127	12	
Critical	25	24	1	
*Age*				
Median (IQR)	60.0 (49.0-68.0)	60.0 (49.0-68.0)	62.0 (50.5-68.0)	0.700
*Gender*				
Male	440	410	30	0.424
Female	627	576	51	
*Hypertension*				
Yes	331	311	20	0.200
No	736	675	61	
*Diabetes*				
Yes	135	126	9	0.664
No	932	860	72	
In hospital				
*Fever*				
Yes(>=37.3°C once or more)	246	228	18	0.853
No	821	758	63	
*Internal visceral dysfunctions*				
Yes	343	313	30	0.327
No	724	673	51	
*Comorbid diseases*				
Yes	560	514	46	0.419
No	507	472	35	
*White blood cell count, ×10^9^* *per L*				
<4	100	94	6	0.579
4-10	927	856	71	
>10	24	21	3	
Unknown	16	15	1	
*Neutrophil count, ×10^9^* *per L*				
<=6.3	994	918	76	1.000
>6.3	57	53	4	
Unknown	16	15	1	
*Lymphocyte count, ×10^9^* *per L*				
<=1.1	158	150	8	0.190
>1.1	894	821	72	
Unknown	15	15	1	
*Platelet count, ×10^9^* *per L*				
<125	44	40	4	0.297
125-350	956	886	69	
>350	52	45	7	
Unknown	15	15	1	
*ALT*				
<40	852	781	71	0.202
>=40	181	171	10	
Unknown	34	34	0	
*Albumin*				
<35	154	144	10	0.502
>=35	880	809	71	
Unknown	33	33	0	
*C-reactive protein*				
<10	914	843	71	0.653
>=10	121	113	8	
Unknown	32	30	2	
*ESR 30min*				
<20	176	163	13	0.420
>=20	282	255	27	
Unknown	609	568	41	
*Procalcitonin*				
<=0.05	589	542	47	0.571
>0.05	207	193	14	
Unknown	271	251	20	
*D-dimer*				
<0.5	507	460	47	0.339
>=0.5	250	232	18	
Unknown	310	294	16	
*BUN*				
<=6.5	853	785	68	0.822
>6.5	148	137	11	
Unknown	66	64	2	
*Creatinine*				
<90	907	839	68	0.150
>=90	94	83	11	
Unknown	66	64	2	
*Accu-Tell Troponin*				
<15.6	590	543	47	1.000
>=15.6	54	50	4	
Unknown	423	393	30	
*IL-6*				0.010
<10	552	509	43	
>=10	63	52	11	
Unknown	452	425	27
Imaging features				
*Consolidation*				
Yes	520	471	49	0.031
No	541	509	32	
Unknown	6	6	0	
*Ground-glass opacity*				
Yes	720	664	56	0.798
No	341	316	25	
Unknown	6	6	0	
*Bilateral pulmonary infiltration*				
Yes	859	789	70	0.193
No	202	191	11	
Unknown	6	6	0	

**Table 3:** Univariate regression analysis for risk factors of patients with recurrence of SARS-CoV-2 RNA positivity.

**Variables**	**No.**	**Univariate OR (95% CI)**	**P value**
Age	1067	1.000(0.985-1.015)	0.995
Gender	1067	1.210(0.757-1.933)	0.425
Clinical severity of disease	1067	0.976(0.579-1.645)	0.927
Hypertension	1067	0.712(0.422-1.200)	0.202
Diabetes	1067	0.853(0.416-1.749)	0.665
Fever	1067	0.950(0.551-1.637)	0.853
Internal visceral dysfunctions	1067	1.265(0.790-2.025)	0.328
Comorbid diseases	1067	1.207(0.764-1.906)	0.420
Neutrophil count, ×10^9^ per L	1051	0.912(0.321-2.587)	0.862
Lymphocyte count, ×10^9^ per L	1051	1.644(0.776-3.484)	**0.194**
Platelet count, ×10^9^ per L	1051	1.417(0.676-2.969)	0.356
ALT	1033	0.643(0.325-1.273)	0.205
Albumin	1034	1.264(0.637-2.508)	0.503
C-reactive protein	1035	0.841(0.394-1.792)	0.653
ESR 30min	458	1.328(0.666-2.647)	0.421
Procalcitonin	796	0.837(0.450-1.553)	0.572
D-dimer	757	0.759(0.431-1.337)	0.340
Accu-Tell Troponin	644	0.924(0.320-2.671)	0.884
IL-6	615	2.504(1.218-5.150)	0.013
Consolidation	1061	1.655(1.042-2.629)	0.033
Ground-glass opacity	1061	1.066(0.653-1.740)	0.798
Bilateral pulmonary infiltration	1061	1.540(0.800-2.966)	**0.196**

**Table 4:** Multivariate regression analysis for risk factors of patients with recurrence of SARS-CoV-2 RNA positivity.

**Variables**	**Multivariate OR (95% CI)**	**P value**
*IL-6*		
<10	Reference	
>=10	3.050(1.432-6.499)	0.004
*Consolidation*		
No	Reference	
Yes	1.641(1.028-2.620)	0.038
*Lymphocyte count, ×10^9^ per L*		
<=1.1	Reference	
>1.1	2.321(1.048-5.138)	0.038
*Bilateral pulmonary infiltration*		
No	Reference	
Yes	1.482(0.764-2.871)	0.244

## DISCUSSION

In this study, we have provided comprehensive data on the demographic and clinical characteristics of 1087 consecutive COVID-19 patients from Wuhan, China. The majority (83.1%) of the cases in our cohort were mild, and the overall mortality rate of the severe and critical cases was 10.6%. The mortality rate of the entire cohort was 1.8%, which is consistent to one previous study [4] but lower than that reported in other studies [5, 12]. This difference can be partly attributed to the higher proportion of severe cases in the other cohorts, as well as the greater medical resources that were allocated in the later stages of this pandemic wherein we enrolled patients for our study. Liang WH et al. [13] reported that the mortality of COVID-19 patients outside of the Hubei Province was limited to 0.3%, as strict public health interventions were initiated in order to prevent further outbreak outside Hubei and adequate medical resources were provided for treatment. In agreement with previous studies that identified older age as a risk factor of mortality in COVID-19 patients [6, 14], the median age of the deceased patients in our cohort was 83 years, distinctly higher than that of the discharged patients (P<0.001), which further suggests that a higher age was significantly associated with mortality.

Among the 1067 patients that were discharged on the basis of negative SARS-CoV-2 RNA results, 81 (7.6%) patients reverted to positive state during their isolation period. Similar findings have been reported previously [11, 15, 16]. However, Yuan J et al. [16] reported a higher repeat positivity rate of 14.5% after discharge, which could be on account the smaller cohort of enrolled patients. These persistent asymptomatic viral carriers may pose a risk for potential future outbreaks despite unprecedented public health interventions [17]. Therefore, we explored the clinical course and risk predictors for recurrent SARS-CoV-2 PCR positivity in order to provide new insights into the disease and help guide the clinical practice against future outbreaks.

In our study, the median duration of viral shedding for patients with positive SARS-CoV-2 RNA recurrence was 33 days from the onset of illness to complete RNA negative status. However, the median duration from illness onset to SARS-CoV-2 RNA reversion was 50 days. Previous studies have reported on duration of viral shedding. Zhou F et al. [6] reported a 20 day median duration of viral shedding in survivors and the longest observed duration was 37 days. Furthermore, Zhou B et al. [18] reported that the median duration of viral shedding was 31 days from illness onset in severe COVID-19 patients. Xu K et al. [19] further showed that 3 out of 4 COVID-19 patients had viral RNA clearance within 21 days of illness onset, and male gender, older age, hypertension, delayed hospital admission, severe illness upon admission, invasive mechanical ventilation and corticosteroid treatment were risk factors for prolonged viral RNA clearance. Our findings underscore the importance of a prolonged treatment or isolation for patients at increased risk of recurrent SARS-CoV-2 RNA positivity.

Nevertheless, we found that age and comorbidities that were previously described to be risk factors of mortality [14] were not identified as significant risk factors when compared to patients without reversion. Instead, high serum IL-6 levels, lymphocyte count greater than 1.1*10^8^ /L and consolidation on CT imaging during hospitalization were associated with a higher likelihood of recurrent SARS-CoV-2 RNA positivity after discharge. This is consistent with a previous study that showed that the lymphocyte count prior to discharge was positively correlated with the time to virus reappearance, which confirms the role of lymphocytes in the potential recurrence of SARS-CoV-2 RNA positivity [16]. Other factors that influence the host defense against viral infections, such as clinical severity of the disease, CRP, D-dimer level etc., were not significantly different between the recurrent versus non-recurrent groups. IL-6 is one of the major pro-inflammatory cytokines that are instrumental in clearing pathogens. However, the rapid multiplication of SARS-CoV-2 in the lower respiratory tract leads to excessive IL-6 production, which triggers an acute severe systemic inflammatory response known as cytokine release syndrome (CRS) [20]. In fact, the increased serum IL-6 levels in severe and critical COVID-19 patients is associated with poor outcomes [21, 22], which was also observed during severe acute respiratory syndrome (SARS) outbreak [23]. Concurrently, lymphopenia is also common in patients with COVID-19, especially in severe and critical cases [5, 22, 24], suggesting a dysregulated immune response in this sub-cohort. In our study however, only 175 (16.1%) patients showed a decrease in lymphocyte count, which again may be can be attributed to the fewer severe cases. Interestingly, the discharged patients with recurrence of positive SARS-CoV-2 RNA had an elevated serum IL-6 level and lymphocyte count compared to those with no recurrence, indicating that the immune system may still be actively involved in clearing the infection. It is also possible that the immune responses can suppress but not completely eradicate SARS-CoV-2, which may have led to the false-negative results due to lower viral loads. Once the virus started replicating again, the RT-PCR results reverted to positive in the discharged patients.

The chest CT imaging of COVID-19 pneumonia is a useful preliminary diagnostic tool that has lowered the rate of missed diagnoses [25]. Features of consolidation on CT imaging are associated with critical disease [26]. Progression of consolidation might indicate further infiltration of the lung parenchyma and lung interstitium due to virus invasion into the respiratory epithelium, which is characterized by diffuse alveolar damage and necrotizing bronchitis. This eventually leads to complete permeation of the alveoli with the inflammatory exudate [27, 28]. Therefore, SARS-CoV-2 may persist in the respiratory epithelium during lung consolidation in the recovery phase of the infection, which eventually results in the recurrence of positive SARS-CoV-2 RNA after discharge. Interestingly, most patients with recurrence had fluctuating positive and negative results in the course of the disease, especially in cases 7, 8 and 41 (Figure 1). This is a potential sign of recurrent SARS-CoV-2 positivity after discharge, and also partly ruled out the randomly error probability in RT-PCR detection for one case. Thus, the infected patients may have already been immune to the virus and require a period for complete recovery. However, if the immune response cannot deal with the recurrence, further treatment may be still needed.

### Limitations

This study has a few limitations that ought to be noted. First, this study was conducted at a single-center hospital which may have introduced a selection bias that influenced the clinical outcomes. A larger multi-center or even global cohort study of COVID-19 patients would help further define the clinical characteristics and risk factors of recurrence. Second, only multipoint throat-swab specimens were tested which increases the risk of false negative results. Therefore, multisite samples should be collected for RT-PCR detection, such as the fecal SARS-CoV-2 RNA test for patients with gastrointestinal symptoms [29]. Third, the retrospective design and initial lack of guidelines for drug administration made it difficult to analyze the impact of treatment regimens on the recurrence of positive SARS-CoV-2 RNA.

## CONCLUSIONS

Elevated lymphocyte counts and serum IL-6 level, and consolidation on chest CT were associated with a greater risk of recurrent SARS-CoV-2 RNA positivity, possibly due to a balance between immune regulation and virus toxicity. For patients with a higher risk of recurrence, a prolonged treatment or isolation period for at least 50 days after illness onset is recommended in order to identify patients that may pose a risk for future outbreaks.

## MATERIALS AND METHODS

### Study design and participants

A total of 1087 consecutive COVID-19 patients diagnosed by SARS-CoV-2 RNA detection in accordance with the interim guidelines of World Health Organization at the Guanggu Branch of Hubei Province Maternity and Childcare Hospital (Wuhan, China) were retrospectively enrolled. All patients had been discharged or had died between February 24, 2020 and March 31, 2020. This study was approved by the Research Ethics Committee of Guanggu Branch of Hubei Province Maternity and Childcare Hospital and was granted with a waiver of informed consent from study participants.

### Data collection and follow-up

The epidemiological, radiographic, laboratory, treatment and treatment outcome data of these patients were extracted from medical records and through direct communication in order to establish a database. The SARS-CoV-2 RNA RT-PCR records from discharge to April 15, 2020 were obtained from the Health Wuhan App, a database containing all real-time results about SARS-CoV-2 RNA tests conducted in Wuhan. The patients were assigned a number for confidentiality. All data were evaluated by two authors (JC and QC) and thereafter by a third researcher (NP) in case of any differences in interpretation.

### Clinical tests

In accordance with the standard procedure, throat-swab specimens were obtained and tested for SARS-CoV-2 infection using RT-PCR by the Academy of Military Medical Sciences and hospital laboratory [14]. The test was repeated during the hospital stay and after clinical remission of symptoms at 24-hour intervals. In addition, serum levels of SARS-CoV-2-specific IgM/IgG measured during hospitalization with the indirect enzyme-linked immunosorbent assay (ELISA) protocol using the N protein of SARS-CoV-2 as the coating antigen. Routine blood tests were performed to determine complete blood counts (including white blood cells, neutrophils, lymphocytes, monocytes and platelets), biochemical indices (liver function, renal function and electrolyte levels), coagulation indices, high-sensitivity C-reactive protein (CRP), erythrocyte sedimentation rate (ESR), procalcitonin, myocardial enzymes, D-dimer and interleukin-6 (IL-6). Computed tomography (CT) scans were routinely performed as recommended by the attending physician.

### Clinical definitions

The patients were discharged based on the following criteria: 1) no fever for at least three days, 2) remission of respiratory symptoms, 3) amelioration of pulmonary inflammation on the chest CT scan, 4) two negative SARS-CoV-2 RNA tests at least 24 hours apart, 5) overall good constitution.

The severity of COVID-19 was defined according to the Chinese management guidelines for COVID-19 (version 6.0) [10]. Fever was defined as axillary temperature of at least 37.3°C. Comorbidities during hospitalization included hypertension, diabetes, hypoproteinaemia (< 25 g/L), coagulopathy (3-second increase in prothrombin time or a 5-second increase of activated partial thromboplastin time), hyperuricemia (blood trioxypurine > 420 μmol/L or > 360 μmol/L in males and females respectively), anemia (according to WHO guidelines [30]), acute respiratory distress syndrome (ARDS; diagnosed according to the Berlin Definition [31]), acute liver failure (diagnosed according to EASL Clinical Practical Guidelines [32]), acute kidney injury (diagnosed according to the KDIGO clinical practice guidelines [33]), and acute cardiac injury (diagnosed as previously reported [6])

### Statistical analysis

Continuous and categorical variables were respectively presented as median with interquartile range (IQR) and counts with percentages. The differences between the recurrence and non-recurrence groups were compared using the Pearson Chis-squared test, Fisher’s exact test or Mann-Whitney U test as appropriate. The risk factors associated with the recurrence of positive SARS-CoV-2 RNA were identified using univariate analysis, and variables with P < 0.2 were selected for multivariate logistic regression model. Missing data was not included in any of the analyses. A two-sided P < 0.05 was considered statistically significant. All statistical analyses were performed using the SPSS v21.0 software (IBM Corporation, Armonk, NY, USA), and the figures were plotted using the GraphPad Prism 8.0 software (GraphPad Software, La Jolla, CA, USA).

## Supplementary Material

Supplementary Table 1
