# Supplementary material for: Clinical course and risk factors for recurrence of positive SARS-CoV-2 RNA: a retrospective cohort study from Wuhan, China
Source: Aging (Albany NY). 2020 Sep 10;12(17):16675–89. doi: 10.18632/aging.103795 (PMC7521537; doi:10.18632/aging.103795)
Supplement: Supplementary Table 1 [file aging-12-103795-s001..pdf]

SUPPLEMENTARY TABLE

Supplementary Table 1. Clinico-demographic characteristics of all patients with COVID-19 confirmed by RT-PCR.

Variables	No. (n=1087)	Percentage (%)
General features		
Clinical severity of disease		
Mild	903	83.1%
Severe	144	13.2%
Critical	40	3.7%
Age		
Median (IQR)	60.0 (49.0-69.0)	
Gender		
Male	452	41.6%
Female	635	58.4%
Hypertension		
Yes	337	31.0%
No	750	69.0%
Diabetes		
Yes	137	12.6%
No	950	87.4%
In hospital		
Fever		
Yes(>=37.3°C once or more)	254	23.4%
No	833	76.6%
Internal visceral dysfunctions		
Yes	363	33.4%
No	724	66.6%
Comorbid diseases		
Yes	580	53.4%
No	507	46.6%
White blood cell count, $\times 10^9$ per L		
<4	102	9.4%
4-10	938	86.3%
>10	31	2.9%
Unknown	16	1.5%
Neutrophil count, $\times 10^9$ per L		
<=6.3	1005	92.5%
>6.3	66	6.1%
Unknown	16	1.5%
Lymphocyte count, $\times 10^9$ per L		
<=1.1	175	16.1%
>1.1	896	82.4%
Unknown	16	1.5%
Platelet count, $\times 10^9$ per L		
<125	52	4.8%
125-350	967	89.0%
>350	52	4.8%
Unknown	16	1.5%
ALT		
<40	867	79.8%
>=40	185	17.0%
Unknown	35	3.2%
Albumin		
<35	171	15.7%
>=35	883	81.2%
Unknown	33	3.0%
C-reactive protein		
<10	919	84.5%
>=10	136	12.5%
Unknown	32	2.9%
ESR 30min		
<20	178	16.4%
>=20	290	26.7%
Unknown	619	56.9%

Procalcitonin		
<=0.05	594	54.6%
>0.05	221	20.3%
Unknown	272	25.0%
D-Dimer		
<0.5	510	46.9%
>=0.5	265	24.4%
Unknown	312	28.7%
BUN		
<=6.5	859	79.0%
>6.5	162	14.9%
Unknown	66	6.1%
Creatinine		
<90	917	84.4%
>=90	104	9.6%
Unknown	66	6.1%
Accu-Tell Troponin		
<15.6	593	54.6%
>=15.6	66	6.1%
Unknown	428	39.4%
IL-6		
<10	555	51.1%
>=10	76	7.0%
Unknown	456	42.0%
IgG		
Positive	887	81.6%
Negative	120	11.0%
Unknown	80	7.4%
IgM		
Positive	797	73.3%
Negative	260	23.9%
Unknown	30	2.8%
Imaging features		
Consolidation		
Yes	525	48.3%
No	551	50.7%
Unknown	11	1.0%
Ground-glass opacity		
Yes	730	67.2%
No	346	31.8%
Unknown	11	1.0%
Bilateral pulmonary infiltration		
Yes	874	80.4%
No	202	18.6%
Unknown	11	1.0%
